# Safety Evaluation of Standardized Extract of *Curcuma longa* (NR-INF-02): A 90-Day Subchronic Oral Toxicity Study in Rats

**DOI:** 10.1155/2021/6671853

**Published:** 2021-07-14

**Authors:** Sasikumar Murugan, Himanshu Solanki, Divya Purusothaman, Bharathi Bethapudi, Mital Ravalji, Deepak Mundkinajeddu

**Affiliations:** ^1^R&D Center, Natural Remedies Private Limited, Bengaluru, Karnataka, India; ^2^Sa-FORD, Navi Mumbai, Maharashtra, India

## Abstract

NR-INF-02 is a standardized extract containing turmerosaccharides from *Curcuma longa* that has anti-inflammatory, analgesic, and chondroprotective potential. In view of its potential uses, NR-INF-02 was evaluated for its safety in Wistar rats at an oral dose of 250, 500, and 1000 mg/kg in a 90-day repeated dose subchronic toxicity study. NR-INF-02 administered at 250, 500, and 1000 mg/kg for 90 days did not show any mortality or clinical signs of toxicity. Body weight gain, food consumption, ocular and neurological examination, and hematological, blood biochemical, hormone, and urine analysis revealed no evidence of toxicity of NR-INF-02 treatment in rats. Absolute and relative organ weights were comparable to control rats. The study did not reveal any major treatment related gross pathological and histopathological alterations in the tissues or organs examined. Thus, based on study observations, the no-observed adverse effect level (NOAEL) was found to be 1000 mg/kg body weight in albino Wistar rats.

## 1. Introduction

Herbal remedy usage is rapidly growing all over the world. In addition, there is growing inclination towards proprietary herbal remedies that are efficacious than regular extracts. However, the safety of the proprietary extracts cannot be relied on its herbal source. Not all the natural treatments are safe and free of adverse effects. Though the efficacy of the herbal remedies is being evinced through conventional experimental methodology, the safety reports of the formulations remain inadequate and meager. The toxicological investigations have to be intensified in compliance with internationally acceptable guideline of safety and toxicity study [1].

A well-known herb *Curcuma longa* of the Zingiberaceae family is native to South and Southeast Asia. It is called as “golden spice” of India. *C. longa* has been reported in Ayurveda, an Indian traditional system of medicine for its effects on wound healing, nausea, indigestion, inflammation, and liver diseases and improving skin complexion. In addition, *C. longa* is extensively researched for fever alleviation and antitumor, antimutagenic, antioxidant, anti-inflammatory, antidiabetic, and other pharmacological effects [2–6]. Most of the effects of *C. longa* have been attributed to curcuminoids. However, the minimally explored aqueous extract of *C. longa* was also found to possess antitumor [7], antidiabetic [8], antimicrobial [9], hepatoprotective [10], fertility-enhancing [11], antidepressant [12], antioxidant [13], antibacterial, and immunomodulatory activities [14]. In the current study, the safety of one such extract, NR-INF-02, developed using a patented aqueous-based process was investigated. NR-INF-02 is a proprietary extract with negligible amount of curcuminoids and is rich in turmerosaccharides. NR-INF-02 is well established as an analgesic [15], anti-inflammatory [16, 17], and antiosteoarthritic agent [15, 18, 19]. NR-INF-02 attenuated the release of chondrocyte-degrading markers like IL-6, IL-8, COX-2, PGE2, tumor necrosis factor-alpha (TNF-*α*), and intercellular adhesion molecule- (ICAM-) 1 in IL-1*β*-treated human knee articular chondrocytes (NHAC-kn). Also, NR-INF-02 protected IL-1*β*-induced degradation of glycosaminoglycans and type II collagen and H_2_O_2_-induced chondrocyte senescence in NHAC-kn cells thereby manifesting to be an antiarthritic agent by maintaining cartilage homeostasis [20]. Likely, NR-INF-02 proved to equilibrate the synthesis and degradation of chondrocyte genes like collagen type II gene, MMP-3, and MMP-7 in osteoarthritis-induced rats [18]. NR-INF-02 showed a significant increase of NO, IL-2, IL-6, IL-10, IL-12, interferon (IFN) gamma, TNF-*α*, and monocyte chemoattractant protein- (MCP-) 1 production in unstimulated mouse splenocytes and mouse macrophages demonstrating its immunomodulatory activity. Interestingly, NR-INF-02 showed its anti-inflammatory activity by exhibiting its potent inhibitory effect towards release of PGE2 and IL-12 levels in lipopolysaccharide-stimulated mouse splenocytes [16].

Pharmacological activities of NR-INF-02 have been evidenced with extensive research. Based on the aforesaid pharmacological studies, NR-INF-02 appears to have huge potential to treat and manage inflammation and pain-related disorders. NR-INF-02 was found to be nonmutagenic through a battery of *in vitro* genotoxicity tests. However, establishing safety of NR-INF-02 becomes quintessential. NR-INF-02 was evaluated for safety in an acute oral toxicity study and found to be safe up to 5 g/kg rat body weight [21]. But acute and genotoxicity data are insufficient to ascertain safety on prolonged consumption. NR-INF-02 was investigated in a subchronic repeated dose toxicity study in rats in accordance to universally acceptable OECD guideline for testing of chemicals to establish NOAEL and to ensure its safety on long-term consumption.

## 2. Materials and Methods

### 2.1. Test Substance

NR-INF-02 is an investigational substance, and its preparation was reported earlier. In brief, rhizomes of turmeric, *C. longa*, were collected from various parts of Tamil Nadu and were authenticated at the National Institute of Science Communication and Information Resource. A voucher specimen (no. 653) was retained at the herbarium of the R&D Center, Natural Remedies Pvt. Ltd., Bengaluru, India. The coarsely powdered rhizomes were steam distilled to remove turmeric oil. The powder was then subjected for water reflux and then concentrated and spray-dried. The spray-dried product and the turmeric oil were blended at the ratio 99 : 1 *w*/*w* to obtain NR-INF-02.

**Figure 1:**
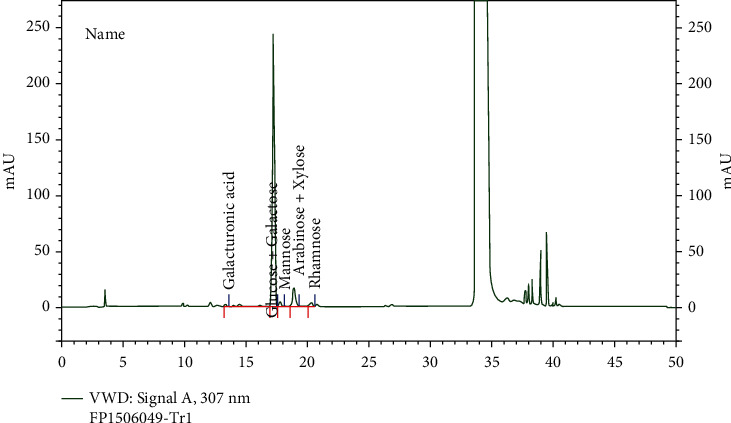
HPLC chromatogram of NR-INF-02.

NR-INF-02 is developed and registered as Turmacin™, a curcumin-free and polysaccharide-enriched water-soluble extract, by Natural Remedies Pvt. Ltd., Bengaluru, India. Characterization of NR-INF-02 was detailed earlier [17]. The total turmerosaccharides in NR-INF-02 were analyzed using HPLC (Shimadzu–LC 2010CHT, Kyoto, Japan), equipped with LiChrospher column C18 (5 *μ*m). Reference standards glucose, galactose, galacturonic acid, mannose, arabinose, xylose, and rhamnose were prepared in HPLC grade water. NR-INF-02 (100 mg) was hydrolysed with 20% trifluoroacetic acid (25 mL), and 10 *μ*L sample was injected. Analysis was carried out at a flow rate of 1 mL/min with ammonium acetate (0.07%) (Solvent A) and acetonitrile (Solvent B) as mobile phase in gradient proportion. The elution was monitored using a UV detector at wavelength 307 nm with a run time of 50 min. HPLC chromatogram of NR-INF-02 was recorded as in Figure 1. Turmerosaccharide content was determined to be >10% *w*/*w*. NR-INF-02 was reported to contain negligible amount of curcumin.

The quality of the product was ensured by subjecting NR-INF-02 for its microbial content (total aerobic microbial count (TAMC), total yeast and mould count (TYMC), bile-tolerant gram-negative bacteria, *E. coli*, *Salmonella species*, and *S. aureus*), aflatoxins, heavy metals, and pesticides as described by the United States Pharmacopoeia (USP) [21] and found to be within the limit as per USP and British Pharmacopoeia [22, 23].

### 2.2. Experimental Procedure

#### 2.2.1. 14-Day Dose Range Finding Study

A dose range finding study was conducted to determine the maximum tolerable dose (MTD) in Wistar rats. Wistar rats of either sex (6-8 weeks) were obtained from the central animal facility, Natural Remedies Pvt. Ltd., Bengaluru, India. The animals were housed in polycarbonate cages provided with laboratory rodent pellet (M/s VRK Nutritional Solutions, Pune, India) with water *ad libitum.* Animals were maintained at room temperature 20-24°C and at relative humidity between 30-70% with a minimum of 12 air changes per hour. 12 : 12 h light-dark cycle was set to maintain circadian rhythm. The potential toxicity data of repeated dose of NR-INF-02 assisted to select the dose level for definitive 90-day subchronic toxicity study. The study was conducted in non-GLP lab in accordance to the committee for the purpose of control and supervision of experiments on animals (CPCSEA) guidelines. Groups consisting 5 male and 5 female rats were administered orally with NR-INF-02 daily once at the dose of 250, 500, and 1000 mg/kg for 14-day. A control group received vehicle (0.5% *w*/*v* CMC) at the dose of 10 mL/kg. Body weight and feed intake were measured at initiation of study and weekly thereafter. During the study, mortality and clinical signs of toxicity were observed daily. On day 15, blood was collected from overnight-fasted rats for hematological and biochemical analysis. Rats were then sacrificed for gross pathological examination, and organs were excised for histopathological examination to determine intoxication in rats.

#### 2.2.2. 90-Day Repeated Dose Subchronic Toxicity Study

Wistar rats of either sex (6-8 weeks) were used for the study. Animals were procured from Global Bioresearch Solution Pvt. Ltd. Pune, India. The animals were housed in polycarbonate cages provided with laboratory rodent pellet (M/s Nutrivet Life Sciences, Pune, India) with water *ad libitum.* Animals were maintained at room temperature 19-24°C and at relative humidity between 30-70% with a minimum of 12 air changes per hour. 12 : 12 h light-dark cycle was set to maintain circadian rhythm. Subchronic toxicity study was approved by IAEC (IAEC proposal no. SF_17_30_021_Extension) and was conducted in accordance to the CPCSEA guidelines in a GLP-certified test facility. The study was conducted in compliance with Schedule Y, Drugs and Cosmetics (II^nd^ amendment) Rules, 2005, Ministry of Health and Family Welfare, Government of India [24], and in accordance to the OECD guideline for the testing of chemicals no. 408 “Repeated Dose 90-Day Oral Toxicity Study in Rodents” (adopted: 27 June 2018) [25].

Sixty male and female Wistar rats were randomly allotted to four groups consisting of 15 male and 15 female rats per group: G I–G IV (main groups). G I, G II, G III, and G IV were administered 0, 250, 500, and 1000 mg/kg of NR-INF-02, respectively. Additional two satellite groups, G IR and G IVR (recovery groups), for control and high dose (1000 mg/kg) having 8 male and 8 female rats per group, were given respective treatment for 90-day and thereafter monitored for further period (recovery period) of 28-day without any treatment to assess reversibility, persistence, or delayed occurrence of toxic effects. G I and G IR were administered with vehicle (0.5% *w*/*v* CMC). The dose volume of 10 mL/kg was kept constant for all dose levels with respect to its most recently recorded body weight. All treatments were administered orally once daily for 90-day.

*(1) Mortality/Morbidity and Clinical Signs*. All animals were observed twice daily for mortality and morbidity throughout the study. Clinical signs were observed before and after dose administration, preferably at the same time each day. Detailed clinical examination was done weekly. Detailed examination includes but not limited to changes in skin, fur, eyes, and mucous membranes; occurrence of secretions and excretions; and autonomic activity (e.g., lacrimation, piloerection, pupil size, and unusual respiratory pattern). Changes in gait, posture, and response to handling as well as the presence of clonic or tonic movements, stereotypes (e.g., excessive grooming and repetitive circling), or bizarre behavior (e.g., self-mutilation and walking backwards) were observed.

*(2) Body Weight and Feed Intake*. Body weight and feed consumption were recorded every week throughout the study period.

*(3) Ophthalmological Examination*. Ocular examination was conducted prior to exposure of animals to the treatment and during week 13 for animals of the main group and on week 17 for animals of the recovery groups. Before examination, mydriasis was induced using 1% tropicamide solution. The examination of the cornea, eyelids, sclera, and conjunctiva for obvious lesions such as chemosis, excess lacrimation, opacity of the cornea, ptosis, chromodacryorrhea, and exopthalmoses was done with unaided eye. The eyes of animals were observed for corneal edema, corneal opacity, cataract, blood in the anterior chamber, and uveitis with the help of an ophthalmoscope (HEINE Optotechnik, Germany).

*(4) Functional/Neurobehavioral Observation*. Home cage observation, handling observation, open-field observation, grip strength (Grip Strength Meter, Columbus Instruments, USA), and motor activity assessment (Opto Varimex 4 Activity Meter, Columbus Instruments, USA) were performed during week 13 for animals of the main groups and week 17 for animals of the recovery groups. As a part of functional observation battery/neurobehavioral observations, other visual parameters like type of posture, gait and mobility, presence or absence of convulsions, salivation, bizarre behavior, tonic/clonic movements, stereotypy, and lacrimation were also considered.

*(5) Clinical Pathology*. On the day of termination, before necropsy, blood from overnight-fasted animals was collected with anticoagulant to perform hematological parameters and with coagulant for biochemical parameters and hormone analysis.

Hematological parameters were estimated by an autoanalyzer (Medonic CA 620 VET, Boule Diagnostics, Spanga, Sweden). The parameters included are hemoglobin (Hb), packed cell volume (PCV), mean corpuscular volume (MCV), mean corpuscular hemoglobin (MCH), mean corpuscular hemoglobin concentration (MCHC), platelet count (PLT), total erythrocyte count (RBC), and total leukocyte count (WBC). Differential leukocyte count (%) was performed on blood smears, using Leishman's stain, prepared from the collected blood. Clotting time was estimated by a coagulation analyzer (Eazy Clot, Robonik Pvt. Ltd., Thane, India).

The serum biochemical parameters were estimated using a clinical chemistry analyzer (Selectra E, Vitalab, Spankeren, Netherlands). The parameters included are alanine aminotransferase (ALT), aspartate aminotransferase (AST), albumin, globulin, blood urea nitrogen, cholesterol, calcium, creatine kinase (CK), creatinine, glucose, gamma-glutamyl transpeptidase (GGT), lactate dehydrogenase (LDH), phosphorus, triglycerides, total protein, total bilirubin, and urea. Electrolytes sodium, potassium, and chloride were detected in the 9180 Electrolyte Analyzer (Roche Diagnostic, Basel, Switzerland).

Serum hormone analysis was detected in an EnSpire multimode reader (PerkinElmer, Massachusetts, USA) using the respective ELISA kit (KINESISDx, CA, USA). The parameters included are total triiodothyronine (T3), thyroxine (T4), and thyroid-stimulating hormone (TSH).

*(6) Urinalysis*. On the night before the termination day, all animals of the main group and the recovery group were fasted and housed in metabolic cages provided with filtered water *ad libitum*. The urine samples were collected and visually observed for color, appearance, and volume of the urine. The other parameters were analyzed using a semi-autoanalyzer (Uriscan Optima II, YD Diagnostics, Republic of Korea), and parameters included are blood/blood cell, bilirubin, urobilinogen, ketone, proteins, nitrites, glucose, and leukocytes.

*(7) Necropsy and Histopathology*. On the day of termination, overnight-fasted rats were weighed and sacrificed under carbon dioxide anaesthesia and were subjected to complete necropsy. The organs like the brain, adrenals, prostate/seminal vesicle (SV) with central gland (CG), prostate, testes/ovaries, epididymides/uterus, heart, liver, kidneys, spleen, thymus, pituitary, and thyroid with parathyroid were isolated, and absolute organ weight and organ weight relative to fasted body weight were recorded. Sperm evaluation was performed for its motility and morphology. All the organs were then preserved in 10% neutral buffered formalin (NBF) except the eyes and testes which were fixed initially using modified Davidson's fluid for 24 h and then with 10% NBF for histopathological studies. Tissues were trimmed after fixation and kept in cassette for further processing. Preservatives were removed from the tissue by subjecting the loaded tissue cassettes for water wash. After removing excess water from tissues with filter paper, tissues were subjected for dehydration (with increasing grades of alcohol), xylene treatment, and wax impregnation processes. Then, 3-5 *μ*m size tissue sections were made using rotary microtome (Leica RM 2255, India) and then stained with hematoxylin and eosin in a Leica Autostainer (Leica Biosystems, India) and observed for the microscopic morphological changes/abnormalities like cellular morphology, no. of cells, size of cells, and arrangement of tissue in comparison with normal histology of that particular organ observed.

### 2.3. Statistical Analysis

Raw data were analyzed using statistical software SPSS 21 (IBM, New York, USA) and Sigma Plot 11.0 (Systat Software, Inc., CA, USA) for the dose range finding study and for the main study, respectively. For the main study groups, ANOVA was applied as the statistical method, while *t*-test was used for the recovery study group data. The values were expressed as mean ± SD. Values outside 95% confidence interval than control are considered statistically significant (*P* < 0.05).

## 3. Results

### 3.1. 14-Day Dose Range Finding Study

All male and female animals of the control and NR-INF-02-treated groups did not show any clinical signs of toxicity and survived till the end of the study period. No differences were observed between the control and treated groups in body weight and hematological and serum biochemical parameters. No treatmentrelated evidence of toxicity was observed in gross pathological examination (data not shown). Hence, maximum tolerable dose was fixed at 1000 mg/kg rat body weight for 90-day repeated dose subchronic toxicity study.

### 3.2. 90-Day Repeated Dose Subchronic Toxicity Study

#### 3.2.1. Mortality/Morbidity and Clinical Signs

No mortality/morbidity and clinical signs of toxicity were observed throughout the treatment period of 90 days and 28 days of posttreatment recovery period.

#### 3.2.2. Body Weight and Feed Intake

**Figure 2:**
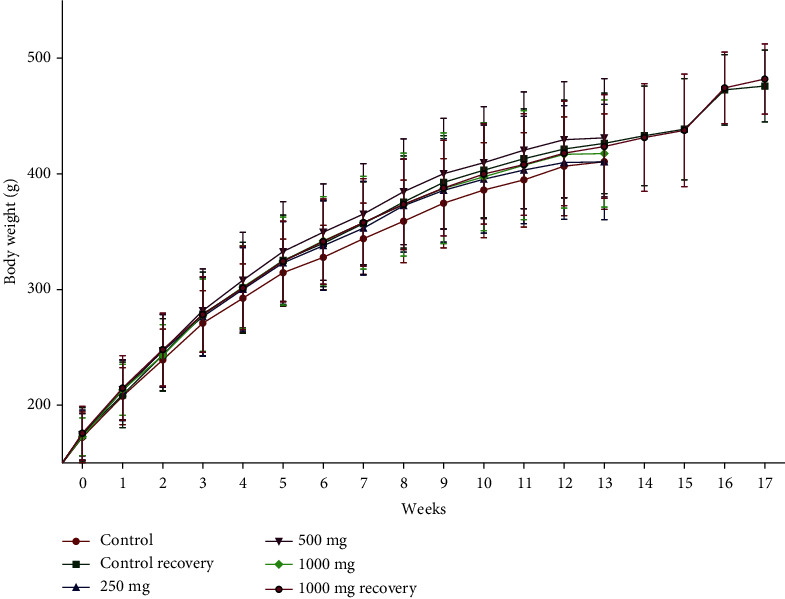
Group mean body weights (mean ± SD) of male rats administered with NR-INF-02 daily for 90 days. *n* = 8 for the recovery groups, and for the remaining groups, *n* = 15.

**Figure 3:**
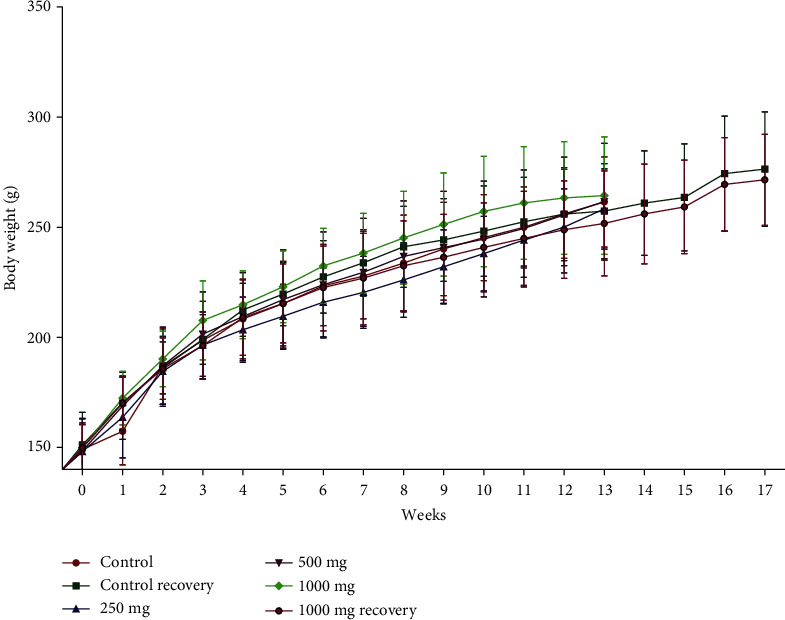
Group mean body weights (mean ± SD) of female rats administered with NR-INF-02 daily for 90 days. *n* = 8 for the recovery groups, and for the remaining groups, *n* = 15.

During the entire experimental period, body weight and body weight gain percentage (percent change in body weight with respect to day 1) of all the treatment groups were comparable with their respective control group except in the female treatment groups (250–1000 mg/kg at week 1). Increased body weight gain percentages 10.45%, 13.74%, and 15.10% were observed in treatment groups at 250, 500, and 1000 mg/kg, respectively, at week 1 compared to the control group. These changes in percent body weight gain were not further observed in the posttreatment recovery group. The data is presented in Figures 2 and 3.

No significant difference was found in feed intake in the treatment group up to 1000 mg/kg in either sex when compared to the control group. Data are presented in Tables 1 and 2.

#### 3.2.3. Ophthalmological Examination

No ophthalmic abnormalities were found in any of the treated groups compared to the control groups throughout the experiment duration.

#### 3.2.4. Functional/Neurobehavioral Observation

No major and consistent differences were observed in the treated groups up to 1000 mg/kg during functional, neurobehavioral observation, grip strength, and motor activity when compared to their control group (Tables S1–S6).

#### 3.2.5. Clinical Pathology

Hematological evaluation showed very few significant differences among the parameters in the treated group when compared to the control group. Significant increase in reticulocytes and decrease in prothrombin time were observed in males of the main group treated with 500 and 1000 mg/kg, while no significant changes were observed in females from any of the treated groups of the main and posttreatment recovery groups when compared with their respective control groups. Data are presented in Tables 3 and 4.

As in hematological evaluation, only very few significant changes were observed in biochemical parameters. In males, significant increase in triglycerides and sodium at the 500 mg/kg treated main group; decrease in phosphorus and globulin and increase in chloride at the 1000 mg/kg posttreated recovery group were observed, while in females, increase in sodium and chloride at the 250 to 1000 mg/kg treated main group and increase in potassium at the 1000 mg/kg treated main group were observed. No such changes were observed in the posttreatment recovery group. The data are presented in Tables 5 and 6.

Evaluation of hormone analysis in males showed significant increase in T3 at 500 mg/kg and TSH at 500 and 1000 mg/kg treated main groups, while in females, significant increase in T4 and TSH at 500 and 1000 mg/kg and significant decrease in T3 at 500 and 1000 mg/kg treated main groups were observed when compared to the control group, whereas no significant changes were observed in T3, T4, and TSH in either sex of the posttreatment recovery group when compared to their respective control group. Data of hormone analysis are given in Tables 7 and 8.

#### 3.2.6. Urinalysis

**Figure 4:**
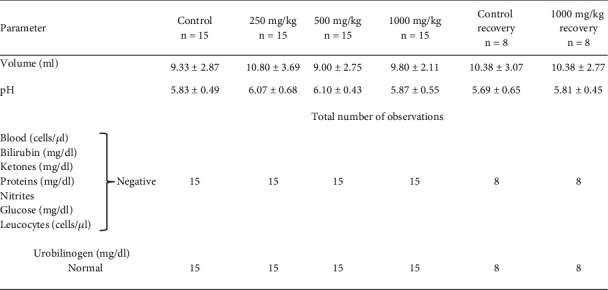
Urine parameters in male rats after oral administration of NR-INF-02 for 90 days. Values of volume and pH are expressed as mean ± SD. Values of other parameters indicate number of animals that fall under respective particulars.

**Figure 5:**
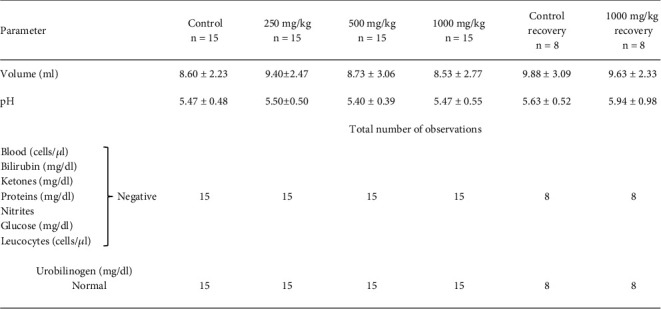
Urine parameters on female rats after oral administration of NR-INF-02 for 90 days. Values of volume and pH are expressed as mean ± SD. Values of other parameters indicate number of animals that fall under respective particulars.

Urinalysis revealed no significant abnormalities in physical, biochemical, and microscopic parameters of urine in either sex of the animals when compared to control. The data is presented in Figures 4 and 5.

#### 3.2.7. Gross Pathology and Organ Weights

No remarkable gross pathological changes were found in the main groups and the recovery groups in either sex of animals treated with different doses of NR-INF-02 in comparison to the control group. In case of organ weights of males, a significant decrease was observed in absolute and relative spleen weight at 250 and 500 mg/kg from the main group and in absolute epididymides weight at the 1000 mg/kg posttreatment recovery group. Significant increase was observed in relative liver weight at the 500 mg/kg treated main group when compared to the control group. In females, significant increase in absolute heart weight and decrease in absolute uterus weight were observed at the 1000 mg/kg posttreatment recovery group when compared to the control group. The data are presented in Tables 9–12.

#### 3.2.8. Histopathology

The histopathology of organs (microscopic observation) showed few pathological findings in either sex of the control group and the group treated with 1000 mg/kg dose. The findings were incidental as the frequency and severity were comparable to their respective control groups and there is a lack of dose relationship. The observations included are lymphocytic infiltration and degeneration in the liver; lymphocytic infiltration in the kidney, lungs, and heart; aneurysm in the aorta; and cytoplasmic vacuolation in the adrenals.

## 4. Discussion

Herbal medicines and herbal healthcare supplements have become an important part of therapy. Because of the definitive adverse effect in modern medicines, herbal remedies reigned more popularly. Traditional medicine and herbal remedies have its history for 5000 years and are based on the theories, beliefs, and experiences indigenous to different cultures used in the prevention, diagnosis, improvement, or treatment of physical and mental illness [26, 27]. Though most of the herbs proved scientifically the traditional pharmacological usage, there were few herbal drugs recently found to be toxic and produce adverse effects. For instance, *Ephedra* has been used widely for the effective treatment for asthma, bronchitis, influenza, etc. and has a history for 5000 years [28]. However, recently in 2004, the U.S. Food and Drug Administration (U.S. FDA) banned the market of supplements and drugs that contain ephedra (ephedrine alkaloid) because of the accumulating adverse effect reports [29]. Following the U.S., many countries including the European Union have restricted the use of ephedra [30]. Likewise, *Datura*, *Aristolochia*, *Piper methysticum*, etc. have been restricted in few countries because of their adverse effects [31, 32]. Thus, many attempts are made at present to harmonize the scientific and regulatory criteria that govern the marketing of herbal products.

In an innovation of herbal supplement for osteoarthritis condition, NR-INF-02 was developed. The present study was conducted to evaluate the in-depth toxicity potential of NR-INF-02. In relevance to understanding the toxicity of the product on long-term oral consumption, a subchronic repeated oral toxicity test was carried out to obtain information on the potential health hazards that may likely occur from continuous exposure, including the information about target organ toxicity, possibilities of cumulative effects, and an estimate of the dose at which there is no observed adverse effect.

Upon the result of the dose range finding study, the oral doses 0, 250, 500, and 1000 mg/kg of NR-INF-02 were selected for repeated dose 90-day oral toxicity study. No mortality or morbidity was observed throughout the study in either sex.

Body weight is a sensitive indicator of health, and study guidelines have considerably emphasized on body weight of the target animal [25]. For toxicity characterization, not more than 20% body weight loss is permitted [33–35]. In this study, increase in percent body weight change was observed only at the first week of the experiment. However, during subsequent weeks, the body weight of the respective group of animals returned to normal and was comparable to control till study completion and also during the posttreatment recovery period. Also, difference in feed intake in male and female rats was considered to be normal as reduced feed intake not less than 75% of the control group was considered to be normal according to the federation of European laboratory animal science associations [36]. Thus, except for incidental increase in body weight at week one, the treatment did not show any changes in body weight and feed intake.

The hematological analysis showed some significant alterations in reticulocytes and prothrombin time at high doses of the NR-INF-02-treated groups. However, the values of these parameters were found to be well within the normal range [37]. As per the OECD guideline, the subchronic repeated oral toxicity study provides toxic information on the target organs. The difference observed in male with respect to absolute and relative splenic weight and relative liver weight was not observed in female rats at the same dose or any other dose levels. Also, the differences observed in males were not dose dependent. Though changes in absolute epididymides weight of males and absolute heart and uterus weight of females from the posttreatment recovery group were observed, no clinical changes were observed in necropsy or related biochemical changes. Thus, NR-INF-02 did not have adverse effects on any specific organ. These observations are in concurrence that the variations were incidental and were not due to the effect of the treatment.

In this study, significant changes in electrolytes like sodium, chloride, and potassium did not demonstrate any significant change during functional or neurobehavioral observation, motor activity, or in urine analysis or in correlated parameters. Also, the values of electrolytes were within the normal range of healthy rats [38]. Likewise, change in values of phosphorus and globulin lied within the normal range. Although few histopathological observations were found, there is a lack of dose relationship, and moreover, these observed lesions are common in occurrence in rodents during toxicological studies [39–41].

In urine analysis, no significant differences in the analyzed parameters between the treated groups and the untreated control were observed.

In case of hormone analysis, in spite of the observed significance increase in T3, T4, and TSH, there were no single event of abnormality observed in the weight and histology of related target organ. It was found that compared to control rats, the NR-INF-02-treated rats did not differ in their absolute and relative thyroid gland weight proving that there is no evident thyroid disruption. To add on, a statistical study report by Beekhuijzen et al. [42] demonstrated that in normal healthy rats the variations of thyroid levels are high, and thus, mere statistical significance cannot be correlated with thyroid dysfunction. Thus, in the light of absence of dose-independent increase or decrease in T3/T4/TSH with inconsistency in males and females and no correlation with body weight or feed intake or organ weight, it proves that values are statistically significant but numerically insignificant. This clearly indicates that NR-INF-02 does not have any toxicological effect on the thyroid. The present *in vivo* study confirmed that the test substance NR-INF-02 is safe over long-term repeated oral consumption, indicating no effects on organs and less possibility of accumulation of test substance and thus established safety criteria for human exposure.

## 5. Conclusion

NR-INF-02 on repeated oral administration in either sex of the rats for 90 days did not demonstrate any significant toxic or adverse effects. The no-observed adverse effect level (NOAEL) of NR-INF-02 in the repeated oral toxicity study was found to be 1000 mg/kg in both male and female animals.

## Figures and Tables

**Table 1:** Group mean feed consumption (g/day/animal) of male rats administered with NR-INF-02 daily for 90 days.

Weeks	Control and control recovery	250 mg/kg		500 mg/kg		1000 mg/kg and 1000 mg/kg recovery	
	Average	Average	% of control	Average	% of control	Average	% of control
1	19.49	19.39	100	19.85	102	20.29	104
2	22.22	22.24	100	23.06	104	23.16	104
3	23.17	22.99	99	24.06	104	23.71	102
4	23.61	23.87	101	25.07	106	23.54	100
5	24.02	23.53	98	24.98	104	23.57	98
6	24.36	23.91	98	25.9	106	24.86	102
7	24.14	23.82	99	24.81	103	24.03	100
8	24.45	24.55	100	26.85	110	25.13	103
9	25.24	24.73	98	26.6	105	25.6	101
10	24.01	23.83	99	24.86	104	24.22	101
11	24.03	23.5	98	25.58	106	22.7	94
12	23.59	22.85	97	24.5	104	22.93	97
13	22.72	19.91	88	20.46	90	20.73	91
14	23.05	—	—	—	—	22.65	98
15	24.16	—	—	—	—	23.24	96
16	24.41	—	—	—	—	23.39	96
17	25.00	—	—	—	**—**	24.89	100
Study average	23.46	23.01	98.06	24.35	103.70	23.42	99.86
Recovery average	24.16					23.54	97.46

**Table 2:** Group mean feed consumption (g/day/animal) of female rats administered with NR-INF-02 daily for 90 days.

Weeks	Control and control recovery	250 mg/kg		500 mg/kg		1000 mg/kg and 1000 mg/kg recovery	
	Average	Average	% of control	Average	% of control	Average	% of control
1	16.48	16.17	98	16.52	100	16.75	102
2	17.13	17.05	100	17.11	100	16.925	99
3	16.94	16.93	100	17.03	101	16.865	100
4	16.69	15.67	94	16.56	99	16.43	98
5	17.11	16.46	96	17.27	101	17.135	100
6	17.36	16.67	96	17.28	100	17.4	100
7	17.37	16.51	95	17.21	99	16.815	97
8	17.17	17.21	100	17.26	101	17.03	99
9	17.40	16.89	97	17.84	103	17.17	99
10	16.52	16.32	99	16.37	99	16.155	98
11	16.93	16.2	96	16.85	100	16.615	98
12	16.54	15.36	93	16.6	100	16.005	97
13	16.04	15.16	95	14.4	90	15.07	94
14	15.74	—	—	—	—	15.91	101
15	16.43	—	—	—	—	16.86	103
16	15.83	—	—	—	—	16.16	102
17	16.30	—	—	—	**—**	16.43	101
Study average	16.90	16.35	96.78	16.79	99.34	16.64	98.49
Recovery average	16.08					16.34	101.64

**Table 3:** Hematological findings (mean ± SD) in male rats after 90 days of subchronic oral administration of NR-INF-02.

Parameter	Control *n* = 15	250 mg/kg *n* = 15	500 mg/kg *n* = 15	1000 mg/kg *n* = 15	Control recovery *n* = 8	1000 mg/kg recovery *n* = 8
RBC (×10^12^/L)	9.05 ± 0.72	8.77 ± 0.48	8.76 ± 0.46	8.65 ± 0.57	8.45 ± 0.61	8.48 ± 0.47
HCT (%)	40.37 ± 2.46	39.15 ± 1.79	38.83 ± 1.51	38.90 ± 2.01	37.93 ± 2.11	38.04 ± 2.08
MCV (fL)	44.65 ± 1.38	44.70 ± 1.66	44.41 ± 2.07	45.01 ± 1.71	44.94 ± 0.97	44.86 ± 0.45
HGB (g/dL)	15.27 ± 0.92	14.91 ± 0.59	14.79 ± 0.55	14.88 ± 0.64	14.30 ± 0.76	14.41 ± 0.70
MCH (pg)	16.90 ± 0.53	17.02 ± 0.69	16.91 ± 0.75	17.25 ± 0.68	16.93 ± 0.42	17.01 ± 0.24
MCHC (g/dL)	37.88 ± 0.38	38.11 ± 0.40	38.09 ± 0.32	38.33 ± 0.59	37.74 ± 0.21	37.94 ± 0.37
PLT (×10^9^/L)	739.80 ± 124.64	747.40 ± 72.78	741.00 ± 76.50	709.67 ± 130.17	734.50 ± 86.59	678.13 ± 56.09
WBC (×10^9^/L)	10.89 ± 2.12	10.30 ± 2.16	10.76 ± 2.41	11.90 ± 5.53	12.84 ± 1.35	11.60 ± 1.17
Neutrophil (%)	18.93 ± 1.75	17.67 ± 1.95	18.33 ± 1.88	17.87 ± 2.07	18.13 ± 2.03	17.38 ± 1.69
Lymphocyte (%)	80.80 ± 1.86	82.00 ± 2.00	81.33 ± 1.99	81.80 ± 1.97	81.50 ± 2.33	82.38 ± 1.77
Monocyte (%)	0.20 ± 0.41	0.27 ± 0.46	0.20 ± 0.41	0.20 ± 0.41	0.25 ± 0.46	0.25 ± 0.46
Eosinophil (%)	0.07 ± 0.26	0.07 ± 0.26	0.13 ± 0.35	0.13 ± 0.35	0.13 ± 0.35	0.00 ± 0.00
Basophil (%)	0.00 ± 0.00	0.00 ± 0.00	0.00 ± 0.00	0.00 ± 0.00	0.00 ± 0.00	0.00 ± 0.00
Reticulocyte (%)	0.87 ± 0.18	1.01 ± 0.16	1.04 ± 0.15^∗^↑	1.05 ± 0.14^∗^↑	1.09 ± 0.18	1.15 ± 0.21
PT (sec.)	24.59 ± 2.21	23.48 ± 1.50	21.89 ± 2.29^∗^↓	22.12 ± 1.30^∗^↓	23.46 ± 2.73	23.29 ± 3.46
aPTT (sec.)	27.90 ± 2.08	28.56 ± 2.85	29.29 ± 2.30	29.95 ± 1.74	25.60 ± 4.43	24.12 ± 5.29

^∗^ *P* < 0.05 versus the control group; ^#^*P* < 0.05 versus the control recovery group; ↑: increase; ↓: decrease.

**Table 4:** Hematological findings (mean ± SD) in female rats after 90 days of subchronic oral administration of NR-INF-02.

Parameter	Control *n* = 15	250 mg/kg *n* = 15	500 mg/kg *n* = 15	1000 mg/kg *n* = 15	Control recovery *n* = 8	1000 mg/kg recovery *n* = 8
RBC (×10^12^/L)	7.70 ± 0.28	7.77 ± 0.31	7.72 ± 0.23	7.84 ± 0.21	7.36 ± 0.25	7.29 ± 0.33
HCT (%)	37.07 ± 1.32	37.57 ± 1.59	37.33 ± 1.00	38.17 ± 1.19	35.95 ± 1.45	35.30 ± 0.96
MCV (fL)	48.13 ± 1.23	48.34 ± 1.55	48.31 ± 1.29	48.67 ± 1.31	48.83 ± 1.22	48.50 ± 1.55
HGB (g/dL)	13.97 ± 0.48	14.16 ± 0.54	14.07 ± 0.35	14.33 ± 0.43	13.58 ± 0.59	13.35 ± 0.36
MCH (pg)	18.16 ± 0.58	18.22 ± 0.46	18.25 ± 0.42	18.29 ± 0.46	18.46 ± 0.44	18.35 ± 0.57
MCHC (g/dL)	37.75 ± 0.79	37.72 ± 0.52	37.77 ± 0.38	37.61 ± 0.50	37.80 ± 0.50	37.86 ± 0.27
PLT (×10^9^/L)	639.07 ± 75.30	666.53 ± 96.89	649.93 ± 91.74	632.73 ± 45.85	692.75 ± 94.14	728.38 ± 93.68
WBC (×10^9^/L)	8.74 ± 2.78	8.35 ± 1.88	7.87 ± 1.90	9.53 ± 2.10	7.43 ± 1.67	7.10 ± 1.76
Neutrophil (%)	17.80 ± 2.01	18.20 ± 2.24	17.80 ± 1.66	18.93 ± 2.19	19.00 ± 2.27	18.50 ± 1.41
Lymphocyte (%)	82.00 ± 2.00	81.53 ± 2.33	81.93 ± 1.75	80.87 ± 2.13	80.50 ± 2.39	81.25 ± 1.39
Monocyte (%)	0.20 ± 0.41	0.20 ± 0.41	0.20 ± 0.41	0.13 ± 0.35	0.38 ± 0.52	0.13 ± 0.35
Eosinophil (%)	0.00 ± 0.00	0.07 ± 0.26	0.07 ± 0.26	0.07 ± 0.26	0.13 ± 0.35	0.13 ± 0.35
Basophil (%)	0.00 ± 0.00	0.00 ± 0.00	0.00 ± 0.00	0.00 ± 0.00	0.00 ± 0.00	0.00 ± 0.00
Reticulocyte (%)	0.97 ± 0.10	0.95 ± 0.06	1.03 ± 0.06	0.97 ± 0.11	1.04 ± 0.09	0.99 ± 0.06
PT (sec.)	22.65 ± 1.60	23.03 ± 1.65	23.86 ± 2.54	23.64 ± 1.92	22.57 ± 2.88	21.05 ± 3.92
aPTT (sec.)	28.33 ± 2.45	29.05 ± 2.19	29.98 ± 1.60	29.69 ± 1.75	22.21 ± 2.75	18.82 ± 3.56

^∗^ *P* < 0.05 versus the control group; ^#^*P* < 0.05 versus the control recovery group; ↑: increase; ↓: decrease.

**Table 5:** Clinical chemistry data (mean ± SD) of male rats after oral administration of NR-INF-02 for 90 days.

Parameter	Control *n* = 15	250 mg/kg *n* = 15	500 mg/kg *n* = 15	1000 mg/kg *n* = 15	Control recovery *n* = 8	1000 mg/kg recovery *n* = 8
ALT (U/L)	84.57 ± 13.60	75.83 ± 12.18	78.55 ± 11.65	77.73 ± 11.59	62.09 ± 7.75	64.99 ± 11.51
AST (U/L)	198.09 ± 39.08	184.89 ± 41.09	205.53 ± 37.89	191.95 ± 29.78	161.43 ± 18.78	148.20 ± 18.45
Albumin (g/dL)	3.78 ± 0.14	3.81 ± 0.25	3.84 ± 0.29	3.77 ± 0.18	3.52 ± 0.24	3.35 ± 0.18
ALP (U/L)	172.71 ± 32.48	145.87 ± 33.28	154.17 ± 42.05	150.68 ± 42.92	152.16 ± 43.96	116.76 ± 32.58
Total protein (g/dL)	7.15 ± 0.19	7.16 ± 0.33	7.12 ± 0.41	6.98 ± 0.28	7.08 ± 0.47	6.64 ± 0.40
Urea (mg/dL)	41.96 ± 4.25	39.48 ± 4.05	42.26 ± 5.24	43.78 ± 3.79	36.56 ± 2.73	38.46 ± 4.71
Bile acid (*μ*mol/L)	16.15 ± 9.84	15.22 ± 9.61	12.59 ± 8.22	13.33 ± 7.91	10.10 ± 3.90	8.54 ± 2.85
Cholesterol (mg/dL)	59.52 ± 10.74	54.85 ± 10.01	66.97 ± 16.49	64.37 ± 8.90	56.68 ± 8.47	60.29 ± 4.09
Calcium (mg/dL)	9.96 ± 0.32	9.93 ± 0.41	10.08 ± 0.45	9.87 ± 0.24	9.87 ± 0.35	9.74 ± 0.34
CK-Nac (U/L)	732.53 ± 351.93	665.27 ± 406.03	887.27 ± 321.75	807.53 ± 354.19	611.13 ± 219.21	534.25 ± 227.59
Creatinine (mg/dL)	0.51 ± 0.04	0.50 ± 0.03	0.49 ± 0.04	0.50 ± 0.03	0.55 ± 0.03	0.55 ± 0.03
Glucose (mg/dL)	79.03 ± 21.28	79.03 ± 14.82	71.40 ± 17.61	81.02 ± 24.21	91.74 ± 17.95	84.68 ± 21.10
GGT (U/L)	1.97 ± 0.80	2.04 ± 1.01	1.70 ± 0.41	1.79 ± 0.69	2.48 ± 0.69	2.12 ± 0.80
LDH (U/L)	2798.27 ± 903.26	2663.47 ± 1303.96	2916.93 ± 742.33	2840.60 ± 778.48	1686.88 ± 682.62	1672.94 ± 531.27
Phosphorus (mg/dL)	5.88 ± 0.55	5.61 ± 0.51	5.82 ± 0.56	5.87 ± 0.37	5.58 ± 0.24	5.31 ± 0.22^#^↓
Triglyceride (mg/dL)	70.52 ± 23.35	76.88 ± 32.91	100.97 ± 28.58^∗^↑	82.43 ± 31.68	84.88 ± 17.60	91.28 ± 13.69
Total bilirubin (mg/dL)	0.39 ± 0.11	0.49 ± 0.27	0.57 ± 0.37	0.44 ± 0.28	0.43 ± 0.15	0.37 ± 0.14
BUN (mg/dL)	19.61 ± 1.99	18.45 ± 1.90	19.75 ± 2.44	20.46 ± 1.77	17.08 ± 1.28	17.98 ± 2.20
A/G ratio	1.13 ± 0.06	1.14 ± 0.10	1.18 ± 0.12	1.18 ± 0.09	0.99 ± 0.05	1.02 ± 0.04
Globulin (g/dL)	3.37 ± 0.14	3.35 ± 0.19	3.28 ± 0.25	3.21 ± 0.23	3.57 ± 0.26	3.30 ± 0.24^#^↓
HDL (mg/dL)	24.21 ± 4.50	22.12 ± 3.70	25.12 ± 4.56	24.87 ± 2.33	20.90 ± 3.74	20.41 ± 2.88
LDL (mg/dL)	21.87 ± 5.62	17.36 ± 5.78	21.67 ± 9.10	24.03 ± 9.07	18.81 ± 6.90	20.65 ± 6.46
Na (mmol/L)	143.80 ± 5.28	145.13 ± 2.36	145.87 ± 2.75^∗^↑	146.07 ± 3.69	142.13 ± 2.10	142.88 ± 1.81
K (mmol/L)	5.51 ± 0.52	5.29 ± 0.52	5.49 ± 0.39	5.31 ± 0.35	4.65 ± 0.32	4.73 ± 0.35
Cl (mmol/L)	101.20 ± 4.54	103.13 ± 2.36	102.67 ± 2.47	103.20 ± 2.31	99.00 ± 1.31	101.25 ± 2.38^#^↑

^∗^ *P* < 0.05 versus the control group; ^#^*P* < 0.05 versus the control recovery group; ↑: increase; ↓: decrease.

**Table 6:** Clinical chemistry data (mean ± SD) of female rats after oral administration of NR-INF-02 for 90 days.

Parameter	Control *n* = 15	250 mg/kg *n* = 15	500 mg/kg *n* = 15	1000 mg/kg *n* = 15	Control recovery *n* = 8	1000 mg/kg recovery *n* = 8
ALT (U/L)	71.93 ± 16.82	66.09 ± 17.09	59.22 ± 15.30	63.32 ± 10.24	57.35 ± 10.38	57.71 ± 12.34
AST (U/L)	192.29 ± 22.17	200.09 ± 42.28	189.12 ± 44.33	221.74 ± 56.24	134.29 ± 27.80	147.39 ± 29.88
Albumin (g/dL)	3.93 ± 0.31	3.86 ± 0.27	3.95 ± 0.20	4.08 ± 0.17	3.74 ± 0.30	3.61 ± 0.21
ALP (U/L)	126.55 ± 46.01	93.41 ± 26.20	113.76 ± 35.11	99.83 ± 28.87	112.48 ± 43.34	102.94 ± 34.30
Total protein (g/dL)	7.77 ± 0.38	7.68 ± 0.62	7.52 ± 0.40	7.98 ± 0.48	7.17 ± 0.44	6.90 ± 0.46
Urea (mg/dL)	47.49 ± 4.98	46.62 ± 9.89	46.55 ± 7.84	49.14 ± 6.51	47.66 ± 8.67	46.89 ± 6.08
Bile acid (*μ*mol/L)	26.77 ± 11.03	24.06 ± 13.48	26.87 ± 20.22	28.67 ± 19.87	26.63 ± 7.73	27.60 ± 6.02
Cholesterol (mg/dL)	64.41 ± 13.07	65.23 ± 13.09	67.14 ± 9.67	71.87 ± 17.59	60.46 ± 10.31	64.63 ± 14.79
Calcium (mg/dL)	10.34 ± 0.53	10.31 ± 0.47	10.45 ± 0.48	10.75 ± 0.45	9.89 ± 0.39	9.83 ± 0.47
CK-Nac (U/L)	638.13 ± 163.73	660.73 ± 256.41	590.00 ± 210.32	614.20 ± 217.69	568.00 ± 166.73	560.75 ± 163.80
Creatinine (mg/dL)	0.52 ± 0.12	0.50 ± 0.08	0.50 ± 0.08	0.48 ± 0.10	0.57 ± 0.04	0.59 ± 0.05
Glucose (mg/dL)	58.07 ± 17.06	67.49 ± 23.56	70.30 ± 25.00	60.57 ± 21.55	97.44 ± 19.99	99.66 ± 12.78
GGT (U/L)	2.71 ± 1.25	2.56 ± 0.69	2.31 ± 0.38	2.48 ± 0.36	2.12 ± 0.82	1.96 ± 0.60
LDH (U/L)	1623.20 ± 497.31	1486.80 ± 1149.81	1472.67 ± 1101.56	1724.53 ± 1045.96	1762.38 ± 290.62	1708.00 ± 344.50
Phosphorus (mg/dL)	5.55 ± 0.70	5.56 ± 0.76	5.63 ± 0.88	5.94 ± 0.63	4.88 ± 0.51	4.36 ± 0.62
Triglyceride (mg/dL)	51.07 ± 16.61	47.93 ± 13.89	55.91 ± 16.58	51.01 ± 15.53	44.98 ± 7.53	52.75 ± 14.66
Total bilirubin (mg/dL)	0.38 ± 0.21	0.32 ± 0.10	0.32 ± 0.06	0.35 ± 0.08	0.45 ± 0.11	0.48 ± 0.09
BUN (mg/dL)	22.20 ± 2.33	21.79 ± 4.63	21.95 ± 3.94	22.96 ± 3.05	22.27 ± 4.04	21.91 ± 2.85
A/G ratio	1.03 ± 0.12	1.03 ± 0.15	1.11 ± 0.11	1.05 ± 0.10	1.09 ± 0.07	1.10 ± 0.07
Globulin (g/dL)	3.84 ± 0.29	3.82 ± 0.54	3.57 ± 0.33	3.91 ± 0.41	3.43 ± 0.18	3.29 ± 0.29
HDL (mg/dL)	27.93 ± 4.34	27.81 ± 4.96	29.27 ± 3.27	28.63 ± 5.52	25.48 ± 3.68	27.65 ± 6.10
LDL (mg/dL)	26.27 ± 7.92	27.85 ± 9.16	26.71 ± 7.09	33.02 ± 12.32	26.01 ± 7.47	26.43 ± 6.93
Na (mmol/L)	139.80 ± 1.21	141.27 ± 3.10^∗^↑	143.00 ± 1.69^∗^↑	143.33 ± 0.82^∗^↑	144.63 ± 1.51	143.13 ± 1.46
K (mmol/L)	4.59 ± 0.48	4.73 ± 0.34	4.71 ± 0.34	5.08 ± 0.42^∗^↑	4.88 ± 0.42	4.71 ± 0.37
Cl (mmol/L)	100.33 ± 1.95	102.60 ± 2.44^∗^↑	103.60 ± 2.41^∗^↑	103.47 ± 1.25^∗^↑	104.00 ± 1.60	105.38 ± 1.19

^∗^ *P* < 0.05 versus the control group; ^#^*P* < 0.05 versus the control recovery group; ↑: increase; ↓: decrease.

**Table 7:** Hormone levels (mean ± SD) of male rats after oral administration of NR-INF-02 for 90 days.

Parameter	T3 (pg/mL)	T4 (ng/mL)	TSH (*μ*IU/mL)
Control	0.21 ± 0.04	7.90 ± 0.70	0.22 ± 0.05
250 mg/kg	0.21 ± 0.04	7.81 ± 2.26	0.27 ± 0.12
500 mg/kg	0.25 ± 0.03^∗^↑	7.48 ± 1.65	0.31 ± 0.08^∗^↑
1000 mg/kg	0.27 ± 0.11	8.69 ± 3.80	0.41 ± 0.10^∗^↑
Control recovery	0.17 ± 0.01	15.32 ± 0.82	0.22 ± 0.02
1000 mg/kg recovery	0.17 ± 0.01	14.16 ± 1.47	0.21 ± 0.03

*n* = 6–15; ^∗^*P* < 0.05 versus the control group; ^#^*P* < 0.05 versus the control recovery group; ↑: increase; ↓: decrease.

**Table 8:** Hormone levels (mean ± SD) of female rats after oral administration of NR-INF-02 for 90 days.

Parameter	T3 (pg/mL)	T4 (ng/mL)	TSH (*μ*IU/mL)
Control	0.74 ± 0.11	8.66 ± 2.07	0.18 ± 0.06
250 mg/kg	0.60 ± 0.29	9.20 ± 2.15	0.22 ± 0.07
500 mg/kg	0.17 ± 0.04^∗^↓	13.29 ± 1.60^∗^↑	0.25 ± 0.03^∗^↑
1000 mg/kg	0.15 ± 0.01^∗^↓	10.17 ± 2.94	0.23 ± 0.04
Control recovery	0.13 ± 0.02	16.87 ± 2.63	0.20 ± 0.03
1000 mg/kg recovery	0.12 ± 0.02	15.45 ± 2.77	0.19 ± 0.03

*n* = 7–15; ^∗^*P* < 0.05 versus the control group; ^#^*P* < 0.05 versus the control recovery group; ↑: increase; ↓: decrease.

**Table 9:** Absolute organ weight (g; mean ± SD) in male rats after oral administration of NR-INF-02 for 90 days.

Parameter	Control *n* = 15	250 mg/kg *n* = 15	500 mg/kg *n* = 15	1000 mg/kg *n* = 15	Control recovery *n* = 8	1000 mg/kg recovery *n* = 8
Body weight	391.55 ± 41.76	390.35 ± 48.85	411.20 ± 51.10	397.08 ± 46.14	460.19 ± 44.95	453.78 ± 30.79
Brain	2.07 ± 0.07	2.01 ± 0.17	2.09 ± 0.06	2.09 ± 0.24	2.09 ± 0.08	2.13 ± 0.12
Adrenals	0.07 ± 0.01	0.07 ± 0.01	0.07 ± 0.01	0.06 ± 0.01	0.07 ± 0.01	0.07 ± 0.01
Prostate/SV gland with CG	2.71 ± 0.50	2.72 ± 0.33	2.82 ± 0.51	2.66 ± 0.41	2.99 ± 0.40	2.54 ± 0.54
Prostate	1.53 ± 0.31	1.45 ± 0.28	1.59 ± 0.49	1.45 ± 0.27	1.56 ± 0.48	1.42 ± 0.37
Testes	3.00 ± 0.46	3.09 ± 0.44	3.30 ± 0.83	3.37 ± 0.58	3.42 ± 0.27	3.15 ± 0.29
Epididymides	1.33 ± 0.20	1.40 ± 0.15	1.47 ± 0.18	1.45 ± 0.17	1.50 ± 0.13	1.33 ± 0.14^#^↓
Heart	1.33 ± 0.19	1.31 ± 0.18	1.40 ± 0.16	1.40 ± 0.19	1.59 ± 0.10	1.48 ± 0.17
Liver	12.55 ± 2.024	12.45 ± 1.87	14.27 ± 2.04	13.32 ± 1.37	15.37 ± 2.03	14.63 ± 2.55
Kidneys	2.73 ± 0.25	2.60 ± 0.37	2.94 ± 0.47	2.87 ± 0.23	3.12 ± 0.25	2.90 ± 0.34
Spleen	0.91 ± 0.16	0.72 ± 0.15^∗^↓	0.75 ± 0.19^∗^↓	0.87 ± 0.11	0.87 ± 0.12	0.82 ± 0.22
Thymus	0.37 ± 0.09	0.34 ± 0.09	0.44 ± 0.15	0.37 ± 0.13	0.40 ± 0.06	0.41 ± 0.13
Pituitary	0.02 ± 0.03	0.01 ± 0.00	0.01 ± 0.00	0.01 ± 0.00	0.02 ± 0.00	0.01 ± 0.00
Thyroid with parathyroid	0.02 ± 0.00	0.02 ± 0.00	0.02 ± 0.00	0.02 ± 0.01	0.03 ± 0.00	0.03 ± 0.00

^∗^ *P* < 0.05 versus the control group. ^#^*P* < 0.05 versus the control recovery group. ↑: increase; ↓: decrease.

**Table 10:** Absolute organ weight (g; mean ± SD) in female rats after oral administration of NR-INF-02 for 90 days.

Parameter	Control *n* = 15	250 mg/kg *n* = 15	500 mg/kg *n* = 15	1000 mg/kg *n* = 15	Control recovery *n* = 8	1000 mg/kg recovery *n* = 8
Body weight	236.70 ± 21.92	228.43 ± 17.71	234.94 ± 26.30	246.22 ± 28.21	260.09 ± 25.26	250.31 ± 18.15
Brain	1.95 ± 0.09	1.89 ± 0.10	1.91 ± 0.13	1.93 ± 0.10	1.87 ± 0.16	1.92 ± 0.08
Adrenals	0.07 ± 0.02	0.07 ± 0.01	0.07 ± 0.01	0.08 ± 0.01	0.08 ± 0.01	0.08 ± 0.02
Ovaries	0.15 ± 0.02	0.15 ± 0.04	0.16 ± 0.02	0.18 ± 0.04	0.14 ± 0.03	0.15 ± 0.03
Uterus	0.45 ± 0.12	0.51 ± 0.27	0.48 ± 0.12	0.47 ± 0.12	0.65 ± 0.13	0.53 ± 0.19^#^↓
Heart	1.08 ± 0.28	0.98 ± 0.26	0.91 ± 0.06	0.98 ± 0.17	0.92 ± 0.11	1.03 ± 0.07^#^↑
Liver	7.51 ± 1.03	7.08 ± 0.76	7.80 ± 1.05	7.85 ± 1.16	7.73 ± 1.05	8.30 ± 0.87
Kidneys	1.66 ± 0.25	1.62 ± 0.19	1.64 ± 0.24	1.76 ± 0.21	1.82 ± 0.14	1.76 ± 0.13
Spleen	0.61 ± 0.19	0.57 ± 0.11	0.60 ± 0.13	0.69 ± 0.17	0.50 ± 0.07	0.54 ± 0.10
Thymus	0.28 ± 0.06	0.30 ± 0.08	0.31 ± 0.07	0.34 ± 0.10	0.28 ± 0.09	0.29 ± 0.05
Pituitary	0.02 ± 0.00	0.01 ± 0.00	0.02 ± 0.00	0.02 ± 0.00	0.02 ± 0.00	0.01 ± 0.00
Thyroid with parathyroid	0.02 ± 0.00	0.02 ± 0.00	0.02 ± 0.00	0.02 ± 0.00	0.02 ± 0.00	0.03 ± 0.00

^∗^ *P* < 0.05 versus the control group. ^#^*P* < 0.05 versus the control recovery group. ↑: increase; ↓: decrease.

**Table 11:** Relative organ weights (% of fasting body weight) (g; mean ± SD) in male rats after oral administration of NR-INF-02 for 90 days.

Parameter	Control *n* = 15	250 mg/kg *n* = 15	500 mg/kg *n* = 15	1000 mg/kg *n* = 15	Control recovery *n* = 8	1000 mg/kg recovery *n* = 8
Brain	0.53 ± 0.05	0.52 ± 0.06	0.52 ± 0.07	0.53 ± 0.08	0.46 ± 0.04	0.47 ± 0.04
Adrenals	0.02 ± 0.00	0.02 ± 0.00	0.02 ± 0.00	0.02 ± 0.00	0.01 ± 0.00	0.01 ± 0.00
Prostate/SV gland with CG	0.69 ± 0.12	0.71 ± 0.12	0.70 ± 0.16	0.68 ± 0.12	0.65 ± 0.06	0.56 ± 0.10
Prostate	0.39 ± 0.09	0.38 ± 0.08	0.39 ± 0.13	0.37 ± 0.08	0.34 ± 0.11	0.32 ± 0.09
Testes	0.77 ± 0.11	0.80 ± 0.13	0.81 ± 0.20	0.86 ± 0.19	0.75 ± 0.07	0.70 ± 0.07
Epididymides	0.34 ± 0.04	0.36 ± 0.04	0.36 ± 0.05	0.37 ± 0.06	0.33 ± 0.04	0.29 ± 0.03
Heart	0.34 ± 0.04	0.34 ± 0.03	0.34 ± 0.03	0.36 ± 0.05	0.35 ± 0.02	0.33 ± 0.03
Liver	3.20 ± 0.37	3.19 ± 0.25	3.47 ± 0.29^∗^↑	3.39 ± 0.47	3.35 ± 0.39	3.22 ± 0.46
Kidneys	0.70 ± 0.05	0.67 ± 0.06	0.72 ± 0.07	0.73 ± 0.11	0.68 ± 0.05	0.64 ± 0.06
Spleen	0.24 ± 0.05	0.19 ± 0.03^∗^↓	0.18 ± 0.05^∗^↓	0.22 ± 0.04	0.19 ± 0.03	0.18 ± 0.04
Thymus	0.09 ± 0.03	0.09 ± 0.02	0.11 ± 0.03	0.09 ± 0.03	0.09 ± 0.01	0.09 ± 0.02
Pituitary	0.01 ± 0.01	0.00 ± 0.00	0.00 ± 0.00	0.00 ± 0.00	0.00 ± 0.00	0.00 ± 0.00
Thyroid with parathyroid	0.00 ± 0.00	0.01 ± 0.00	0.01 ± 0.00	0.01 ± 0.00	0.01 ± 0.00	0.01 ± 0.00

^∗^ *P* < 0.05 versus the control group. ^#^*P* < 0.05 versus the control recovery group. ↑: increase; ↓: decrease.

**Table 12:** Relative organ weights (% of fasting body weight) (g; mean ± SD) in female rats after oral administration of NR-INF-02 for 90 days.

Parameter	Control *n* = 15	250 mg/kg *n* = 15	500 mg/kg *n* = 15	1000 mg/kg *n* = 15	Control recovery *n* = 8	1000 mg/kg recovery *n* = 8
Brain	0.83 ± 0.05	0.83 ± 0.08	0.82 ± 0.07	0.79 ± 0.07	0.73 ± 0.08	0.77 ± 0.05
Adrenals	0.03 ± 0.01	0.03 ± 0.01	0.03 ± 0.01	0.03 ± 0.00	0.03 ± 0.01	0.03 ± 0.01
Ovaries	0.07 ± 0.01	0.07 ± 0.01	0.07 ± 0.01	0.07 ± 0.01	0.06 ± 0.01	0.06 ± 0.01
Uterus	0.19 ± 0.05	0.22 ± 0.11	0.20 ± 0.04	0.20 ± 0.06	0.25 ± 0.05	0.22 ± 0.08
Heart	0.46 ± 0.14	0.44 ± 0.14	0.39 ± 0.04	0.40 ± 0.04	0.36 ± 0.07	0.41 ± 0.03
Liver	3.17 ± 0.32	3.11 ± 0.34	3.33 ± 0.35	3.18 ± 0.18	3.01 ± 0.53	3.31 ± 0.21
Kidneys	0.70 ± 0.06	0.71 ± 0.09	0.70 ± 0.05	0.72 ± 0.06	0.71 ± 0.11	0.70 ± 0.04
Spleen	0.26 ± 0.06	0.25 ± 0.05	0.26 ± 0.05	0.28 ± 0.07	0.20 ± 0.05	0.22 ± 0.04
Thymus	0.12 ± 0.02	0.13 ± 0.03	0.13 ± 0.03	0.14 ± 0.04	0.11 ± 0.04	0.12 ± 0.02
Pituitary	0.01 ± 0.00	0.01 ± 0.00	0.01 ± 0.00	0.01 ± 0.00	0.01 ± 0.00	0.01 ± 0.00
Thyroid with parathyroid	0.01 ± 0.00	0.01 ± 0.00	0.01 ± 0.00	0.01 ± 0.00	0.01 ± 0.00	0.01 ± 0.00

^∗^ *P* < 0.05 versus the control group. ^#^*P* < 0.05 versus the control recovery group. ↑: increase; ↓: decrease.

## Data Availability

Data availability statement will be provided by authors on request.

## Supplementary Materials

Supplementary Materials Table S1: effect on motor activity of male rats after 90 days of the oral administration of NR-INF-02. Table S2: effect on motor activity of female rats after 90 days of the oral administration of NR-INF-02. Table S3: effect of NR-INF-02 in male rats on functional observation battery/neurobehavioral observation after 90 days of oral administration. Table S4: effect of NR-INF-02 in female rats on functional observation battery/neurobehavioral observation after 90 days of oral administration. Table S5: effect of NR-INF-02 in male rats on grip strength after 90 days oral administration. Table S6: effect of NR-INF-02 in female rats on grip strength after 90 days of oral administration.
