# Supplementary material for: Safety Evaluation of Standardized Extract of Curcuma longa (NR-INF-02): A 90-Day Subchronic Oral Toxicity Study in Rats
Source: Biomed Res Int. 2021 Jul 14;2021:6671853. doi: 10.1155/2021/6671853 (PMC8294958; doi:10.1155/2021/6671853)
Supplement: Supplementary Materials — Table S1: effect on motor activity of male rats after 90 days of the oral administration of NR-INF-02. Table S2: effect on motor activity of female rats after 90 days of the oral administration of NR-INF-02. Table S3: effect of NR-INF-02 in male rats on functional observation battery/neurobehavioral observation after 90 days of oral administration. Table S4: effect of NR-INF-02 in female rats on functional observation battery/neurobehavioral observation after 90 days of oral administration. Table S5: effect of NR-INF-02 in male rats on grip strength after 90 days oral administration. Table S6: effect of NR-INF-02 in female rats on grip strength after 90 days of oral administration. [file 6671853.f1.docx]

| **Parameter** | **Control^@@^**  **n=15** | **250 mg/kg^@@^**  **n=15** | **500 mg/kg^@@^**  **n=15** | **1000 mg/kg^@@^**  **n=15** | **Control recovery^@^**  **n=08** | **1000 mg/kg recovery^@^**  **n=08** |
| --- | --- | --- | --- | --- | --- | --- |
| DT (cm) | 2052.60 ± 487.99 | 1945.60 ± 457.33 | 2064.00 ± 656.64 | 1577.20±342.87↓***** | 1150.00±347.38 | 2228.63±451.07 |
| RT (sec) | 136.60 ± 33.05 | 136.87 ± 45.57 | 136.87 ± 43.25 | 176.13±40.61 ↑* | 227.00±59.59 | 128.75±33.77 |
| ST (sec) | 143.87 ± 13.90 | 129.87 ± 22.28 | 147.20 ± 23.42 | 147.60±18.50 | 148.88±20.61 | 131.50±27.88 |
| AT (sec) | 319.53 ± 40.49 | 333.27 ± 55.20 | 315.93 ± 56.69 | 276.27±49.17 | 224.13±57.61 | 339.75±39.37 |
| BSM | 94.60 ± 8.01 | 92.40 ± 12.21 | 95.20 ± 10.80 | 95.80±7.42 | 98.63±8.88 | 91.63±15.04 |
| HC | 1771.33 ± 361.39 | 1661.13 ± 308.93 | 1837.27 ± 523.48 | 1437.33±264.49↓***** | 1096.63±268.59 | 1919.50±307.26 |
| AC | 1187.87 ± 313.75 | 1100.33 ± 244.38 | 1231.13 ± 432.99 | 913.20 ± 219.28↓***** | 662.38±206.05 | 1304.63±281.21 |
| HB | 0.93 ± 0.26 | 1.07 ± 0.59 | 1.07 ± 0.59 | 1.00±0.00 | 0.88±1.13 | 1.00±0.53 |
| VC | 0.00 ± 0.00 | 0.00 ± 0.00 | 0.00 ± 0.00 | 0.00 ± 0.00 | 0.00 ± 0.00 | 0.00 ± 0.00 |
| VB | 0.00 ± 0.00 | 0.00 ± 0.00 | 0.00 ± 0.00 | 0.00 ± 0.00 | 0.00 ± 0.00 | 0.00 ± 0.00 |
| CR | 26.33 ± 4.25 | 30.13 ± 6.47 | 30.07±9.90 | 22.40±4.79 | 17.50±4.69 | 32.00±6.05 |
| CCR | 29.67 ± 6.25 | 27.60 ± 6.57 | 27.07±9.46 | 23.73±7.05 | 18.25±5.18 | 31.13±7.68 |
| V2C | 0.00 ± 0.00 | 0.00 ± 0.00 | 0.00±0.00 | 0.00 ± 0.00 | 0.00 ± 0.00 | 0.00±0.00 |
| V2B | 0.00 ± 0.00 | 0.00 ± 0.00 | 0.00±0.00 | 0.00 ± 0.00 | 0.00 ± 0.00 | 0.00±0.00 |

Table S1: Effect on motor activity of male rats after 90 days of the oral administration of NR-INF-02

cm=Centimeter, sec=Second, DT=Distance travelled, RT=Resting time, ST=Stereotypic time, AT=Ambulatory time, BSM= Burst of stereotypic movements, HC= Horizontal counts, AC=Ambulatory count, HB=Horizontal break, VC = Vertical sensor counts, VB = Vertical sensor breaks, CR=Clockwise rotation, CCR=Counter clockwise rotation, V2C = Second vertical sensor counts, V2B = Second vertical sensor breaks, ↑* = Statistically significant increase (p < 0.05), ↓***** = Statistically significant decrease (p < 0.05). **^@@^** Data related to Week 13; ^@^ Data related to Week 17.

Table S2: Effect on motor activity of female rats after 90 days of the oral administration of NR-INF-02

| **Parameter** | **Control^@@^**  **n=15** | **250 mg/kg^@@^**  **n=15** | **500 mg/kg^@@^**  **n=15** | **1000 mg/kg^@@^**  **n=15** | **Control recovery^@^**  **n=08** | **1000 mg/kg recovery^@^**  **n=08** |
| --- | --- | --- | --- | --- | --- | --- |
| DT (cm) | 2020.93±842.44 | 2915.00±961.77↑* | 2875.53±363.41↑* | 2597.27±552.22 | 3249.00±1389.27 | 2229.00±332.98 |
| RT (sec) | 212.47±91.19 | 127.60±25.59↓***** | 120.13±22.03↓***** | 139.13±34.31 | 116.25±30.63 | 132.50±32.49 |
| ST (sec) | 133.60±29.26 | 132.80±24.68 | 127.67±12.38 | 131.20±23.06 | 120.00±17.95 | 143.75±16.25 |
| AT (sec) | 253.93±80.55 | 339.60±43.13↑* | 352.20±24.87↑* | 329.67±48.62 | 363.75±39.26 | 323.75±30.61 |
| BSM | 90.93±15.48 | 90.93±13.81 | 91.07±8.46 | 88.80±10.09 | 83.75±12.89 | 97.63±6.37 |
| HC | 1737.87±659.40 | 2378.93±741.96↑* | 2331.00±291.63↑* | 2136.67±493.40 | 2581.13±1326.77 | 1876.50±240.11 |
| AC | 1203.27±545.17 | 1715.87±657.82 | 1689.47±226.47↑* | 1525.33±430.36 | 1922.13±1243.45 | 1291.88±217.86 |
| HB | 0.87±0.35 | 1.13±0.64 | 1.00±0.00 | 1.20±0.77 | 1.38±1.19 | 1.00±0.00 |
| VC | 0.00 ± 0.00 | 0.00 ± 0.00 | 0.00 ± 0.00 | 0.00 ± 0.00 | 0.00 ± 0.00 | 0.00 ± 0.00 |
| VB | 0.00 ± 0.00 | 0.00 ± 0.00 | 0.00 ± 0.00 | 0.00 ± 0.00 | 0.00 ± 0.00 | 0.00 ± 0.00 |
| CR | 22.87±7.95 | 33.53±9.04↑* | 33.13±6.36↑* | 32.73±4.93↑* | 38.88±5.38 | 28.13±5.96 |
| CCR | 26.87±10.89 | 33.33±7.88 | 33.60±7.78 | 27.67±10.57 | 37.13±14.37 | 27.38±4.84 |
| V2C | 0.00 ± 0.00 | 0.00 ± 0.00 | 0.00±0.00 | 0.00 ± 0.00 | 0.00 ± 0.00 | 0.00±0.00 |
| V2B | 0.00 ± 0.00 | 0.00 ± 0.00 | 0.00±0.00 | 0.00 ± 0.00 | 0.00 ± 0.00 | 0.00±0.00 |

cm=Centimeter, sec=Second, DT=Distance travelled, RT=Resting time, ST=Stereotypic time, AT=Ambulatory time, BSM= Burst of stereotypic movements, HC= Horizontal counts, AC=Ambulatory count, HB=Horizontal break, VC = Vertical sensor counts, VB = Vertical sensor breaks, CR=Clockwise rotation, CCR=Counter clockwise rotation, V2C = Second vertical sensor counts, V2B = Second vertical sensor breaks, ↑* = Statistically significant increase (p < 0.05), ↓***** = Statistically significant decrease (p < 0.05). **^@@^** Data related to Week 13; ^@^ Data related to Week 17.

Table S3: Effect of NR-INF-02 in male rats on Functional observation battery/neurobehavioral observation after 90D oral administration

| Parameter | Observation | Control n=15^@@^ | 250 mg/kg n=15^@@^ | 500 mg/kg n=15^@@^ | 1000 mg/kg n=15^@@^ | Control recovery n=8^@^ | 1000 mg/kg recovery n=8^@^ |
| --- | --- | --- | --- | --- | --- | --- | --- |
|  |  | Number of animals showing observation | | | | | |
| Posture | Curled up, often sleep | 15 | 15 | 15 | 15 | 8 | 6 |
|  | Sleep | 0 | 0 | 0 | 0 | 0 | 2 |
| Convulsions | Absent | 15 | 15 | 15 | 15 | 8 | 8 |
| Ease of removal from the cage | Very easy | 15 | 15 | 15 | 15 | 8 | 8 |
| Handling reactivity | Easy | 15 | 15 | 15 | 15 | 8 | 8 |
| Palpebral closure | Eyelids wide open | 15 | 15 | 15 | 15 | 8 | 8 |
| Lacrimation | No lacrimation | 15 | 15 | 15 | 15 | 8 | 8 |
| Piloerection | Absent | 15 | 15 | 15 | 15 | 8 | 8 |
| Skin examination | Absent | 15 | 15 | 15 | 15 | 8 | 8 |
| Salivation | No salivation | 15 | 15 | 15 | 15 | 8 | 8 |
| Gait | Normal | 15 | 15 | 15 | 15 | 8 | 8 |

^@@^ Week 13; ^@^Week 17.

Table S3: Effect of NR-INF-02 in male rats on Functional observation battery/neurobehavioral observation after 90D oral administration (contd…)

| Parameter | Observation | Control n=15^@@^ | 250 mg/kg n=15^@@^ | 500 mg/kg n=15^@@^ | 1000 mg/kg n=15^@@^ | Control recovery n=8^@^ | 1000 mg/kg recovery n=8^@^ |
| --- | --- | --- | --- | --- | --- | --- | --- |
|  |  | Number of animals showing observation | | | | | |
| Mobility | Normal | 15 | 15 | 15 | 15 | 8 | 8 |
| Arousal | Normal | 15 | 15 | 15 | 15 | 8 | 8 |
| Respiration | Normal | 15 | 15 | 15 | 15 | 8 | 8 |
| Tonic movement | Absent | 15 | 15 | 15 | 15 | 8 | 8 |
| Clonic movement | Absent | 15 | 15 | 15 | 15 | 8 | 8 |
| Stereotypy | Normal | 15 | 15 | 15 | 15 | 8 | 8 |
| Bizarre behaviour | Absent | 15 | 15 | 15 | 15 | 8 | 8 |
| No. of rears | Mean±SD | 8.73±1.16 | 8.93±1.16 | 9.00±1.13 | 8.40±0.91 | 9.13±1.73 | 9.50±2.14 |
| Vocalization count | Mean±SD | 0.00±0.00 | 0.00±0.00 | 0.00±0.00 | 0.00±0.00 | 0.00±0.00 | 0.00±0.00 |
| No. of urine pools | Mean±SD | 3.53±1.30 | 3.73±0.96 | 3.27±0.96 | 1.53±1.19↓***** | 2.00±1.31 | 2.63±0.92 |
| No. of faecal bolus | Mean±SD | 2.07±1.39 | 1.87±1.36 | 1.53±1.19 | 0.67±1.05↓***** | 1.63±1.77 | 2.25±1.39 |

∗p < 0.05 versus control group; ↓ - decrease; ^@@^ Week 13; ^@^Week 17.

Table S4: Effect of NR-INF-02 in female rats on Functional observation battery/neurobehavioral observation after 90D oral administration

| Parameter | Observation | Control n=15^@@^ | 250 mg/kg n=15^@@^ | 500 mg/kg n=15^@@^ | 1000 mg/kg n=15^@@^ | Control recovery n=8^@^ | 1000 mg/kg recovery n=8^@^ |
| --- | --- | --- | --- | --- | --- | --- | --- |
|  |  | Number of animals showing observation | | | | | |
| Posture | Curled up, often sleep | 12 | 13 | 13 | 15 | 7 | 6 |
|  | Sitting B | 3 | 2 | 2 | 0 | 1 | 2 |
| Convulsions | Absent | 15 | 15 | 15 | 15 | 8 | 8 |
| Ease of removal from the cage | Very easy | 15 | 15 | 15 | 15 | 8 | 8 |
| Handling reactivity | Easy | 15 | 15 | 15 | 15 | 8 | 8 |
| Palpebral closure | Eyelids wide open | 15 | 15 | 15 | 15 | 8 | 8 |
| Lacrimation | No lacrimation | 15 | 15 | 15 | 15 | 8 | 8 |
| Piloerection | Absent | 15 | 15 | 15 | 15 | 8 | 8 |
| Skin examination | Absent | 15 | 15 | 15 | 15 | 8 | 8 |
| Salivation | No salivation | 15 | 15 | 15 | 15 | 8 | 8 |
| Gait | Normal | 15 | 15 | 15 | 15 | 8 | 8 |

^@@^ Week 13; ^@^Week 17.

Table S4: Effect of NR-INF-02 in female rats on Functional observation battery/neurobehavioral observation after 90D oral administration (contd…)

| Parameter | Observation | Control n=15^@@^ | 250 mg/kg n=15^@@^ | 500 mg/kg n=15^@@^ | 1000 mg/kg n=15^@@^ | Control recovery n=8^@^ | 1000 mg/kg recovery n=8^@^ |
| --- | --- | --- | --- | --- | --- | --- | --- |
|  |  | Number of animals showing observation | | | | | |
| Mobility | Normal | 15 | 15 | 15 | 15 | 8 | 8 |
| Arousal | Normal | 15 | 15 | 15 | 15 | 8 | 8 |
| Respiration | Normal | 15 | 15 | 15 | 15 | 8 | 8 |
| Tonic movement | Absent | 15 | 15 | 15 | 15 | 8 | 8 |
| Clonic movement | Absent | 15 | 15 | 15 | 15 | 8 | 8 |
| Stereotypy | Normal | 15 | 15 | 15 | 15 | 8 | 8 |
| Bizarre behaviour | Absent | 15 | 15 | 15 | 15 | 8 | 8 |
| No. of rears | Mean±SD | 9.67±0.18 | 8.73±2.69 | 9.67±1.35 | 8.33±2.38 | 10.88±2.23 | 10.75±2.25 |
| Vocalization count | Mean±SD | 0.00±0.00 | 0.00±0.00 | 0.00±0.00 | 0.00±0.00 | 0.00±0.00 | 0.00±0.00 |
| No. of urine pools | Mean±SD | 0.60±0.63 | 1.60±1.18 | 1.00±1.20 | 2.13±1.81↑* | 1.00±0.25 | 0.75±0.89 |
| No. of faecal bolus | Mean±SD | 0.40±0.91 | 0.53±0.74 | 0.60±1.24 | 0.33±0.62 | 0.25±0.46 | 0.25±0.46 |

∗p < 0.05 versus control group; ↑ - increase; ^@@^ Week 13; ^@^Week 17.

Table S5: Effect of NR-INF-02 in male rats on grip strength after 90D oral administration

| Parameter | Control^@@^  n=15 | 250 mg/kg^@@^  n=15 | 500 mg/kg^@@^  n=15 | 1000 mg/kg^@@^  n=15 | Control recovery^@^  n=8 | 1000 mg/kg recovery^@^  n=8 |
| --- | --- | --- | --- | --- | --- | --- |
| Fore limb grip strength (g) | 882±39.24 | 859.62±42.72 | 861.73±51.75 | 803.87±37.59↓***** | 785.50±59.34 | 779.96±29.73 |
| Fore limb grip strength (g) | 481.38±35.00 | 502.62±43.32 | 492.60±43.38 | 510.04±39.37 | 513.50±36.77 | 477.83±28.71 |

∗p < 0.05 versus control group; ↓ - decrease; ^@@^ Week 13; ^@^Week 17.

Table S6: Effect of NR-INF-02 in female rats on grip strength after 90D oral administration

| Parameter | Control^@@^  n=15 | 250 mg/kg^@@^  n=15 | 500 mg/kg^@@^  n=15 | 1000 mg/kg^@@^  n=15 | Control recovery^@^  n=08 | 1000 mg/kg recovery^@^  n=08 |
| --- | --- | --- | --- | --- | --- | --- |
| Fore limb grip strength (g) | 778.80±31.23 | 768.76±28.46 | 776.53±23.53 | 795.71±33.62 | 748.83±41.25 | 756.38±78.88 |
| Fore limb grip strength (g) | 470.56±43.80 | 463.24±30.14 | 506.33±30.14 | 493.27±24.02 | 526.67±30.06 | 528.21±42.23 |

^@@^ Week 13; ^@^Week 17
